# Genetic Analysis of the IncX4 Plasmids: Implications for a Unique Pattern in the *mcr-1* Acquisition

**DOI:** 10.1038/s41598-017-00095-x

**Published:** 2017-03-24

**Authors:** Jian Sun, Liang-Xing Fang, Zuowei Wu, Hui Deng, Run-Shi Yang, Xing-Ping Li, Shu-Min Li, Xiao-Ping Liao, Youjun Feng, Ya-Hong Liu

**Affiliations:** 1 National Risk Assessment Laboratory for Antimicrobial Resistance of Animal Original Bacteria, South China Agricultural University, Guangzhou, People’s Republic of China; 2 Guangdong Provincial Key Laboratory of Veterinary Pharmaceutics Development and Safety Evaluation, South China Agricultural University, Guangzhou, People’s Republic of China; 3 Department of Veterinary Microbiology and Preventive Medicine, College of Veterinary Medicine, Iowa State University, Ames, USA; 4 Department of Medical Microbiology and Parasitology, Zhejiang University School of Medicine, Zhejiang, 310058 People’s Republic of China

## Abstract

IncX4 plasmids are associated with the dissemination of the *mcr-1* genes in Enterobacteriaceae. We screened IncX4 plasmids among 2,470 isolates of Enterobacteriaceae and determined the *mcr-1* positive isolates. Forty-three isolates were observed to carry IncX4 type plasmid, among which 13 were identified to carry *mcr-1* gene. Three representative *mcr-1*-positive IncX4 plasmids were selected for high-throughput sequencing. Comparative genomics showed that the *mcr-1*-carrying IncX4 plasmids exhibit remarkable similarity in the backbone, and the major distinction lies in the region containing *mcr-1*. The major variable regions of all the IncX4 plasmids were fully characterized by PCR-RFLP. The results revealed that the *mcr-1* was located on the Variable Region I of IncX4 plasmids in 11 *E. coli* isolates. Among them, nine *E. coli* strains possess an epidemic pCSZ4-like IncX4 plasmid containing *mcr-1*. IS*Apl1* was presumably involved in the transposition of the *mcr-1* cassette and then was lost. Similar genetic contexts were found in different plasmids, even the *E. coli* chromosome, implying the acquisition of *mcr-1* by a unique common mechanism.

## Introduction

Plasmid-mediated gene horizontal transfer plays an important role in the dissemination of antibiotic resistance determinants in Gram-negative bacteria^1^. IncX plasmid is a narrow-host range plasmid of Enterobacteriaceae, and exists as a resident plasmid since the pre-antibiotic era^2^. Plasmid relaxase gene typing (PRaseT), suggested that IncX plasmids can be grouped into six members (from IncX1, IncX2, …, IncX6), which are frequently relevant to the spread of the antibiotic resistance genes like fluoroquinolone and β-lactam resistance^3, 4^.

Among them, IncX4 is one of the most prevalent plasmid type in *E. coli* (7.6% to 34.0%)^3, 5^. Also, IncX4 plasmids are found in other species of Enterobacteriaceae^3–6^. IncX4 plasmid is self-transferable at high frequencies (~10^−1^–~10^−4^), which is much higher (10^2^–10^5^ -fold) than the epidemic IncFII plasmids^5, 7^. In particular, the dissemination of the gene *bla*_CTX-M-14b_ by the IncX4 type plasmid pSAM7^8^ was observed amongst the following three species (*E. coli*, *Enterobacter cloacae*, and *Salmonella enterica)*. It suggested that IncX4 plasmids also can be transferred between different species of Enterobacteriaceae.

Since its first discovery in China, in the later of 2015^9^, the *mcr-1* colistin resistance gene has been reported worldwide encompassing all continents except Oceania and Antarctica^10, 11^. Diversified plasmids act as major vectors for the dissemination of the *mcr-1* gene in Enterobacteriaceae^12, 13^. So far, the *mcr-1*-harbouring plasmids can be grouped into eight types, including IncI2, IncX4, IncHI1, IncHI2, IncF, IncFI, IncFII, and IncP^9, 14–21^. As a prevalent type, IncX4 plasmid is widespread in various species (*E. coli*, *Salmonella enteric*, and *Klebsiella pneumonia*) of diversified origins ranging from human, animals, to animal products in many countries, like China, Denmark, United Kingdom, etc.^15–17, 22–29^. Of being noteworthy, the *mcr-1*-carring IncX4 plasmids can occur in extended-spectrum β-lactamase (ESBL) - and carbapenemase- producing Enterobacteriaceae^22, 23^, posing severe threat to public health.

Generally, the *mcr-1* gene is present within the approximately 2,600 bp long fragment designed as the *mcr-1* cassette in which the *mcr-1* gene is followed by a hypothetical protein annotated with the phosphoesterase^29^. IS*Apl1* upstream of the *mcr-1* gene, presumably accounts for the mobilization of the *mcr-1* gene^30^. Occasionally, the *mcr-1* gene exists in a composite transposon having the boundaries with two copies of IS*Apl1* ^21^. Recently, a model for transposition of *mcr-1* by IS*Apl1* has been illustrated^31^. But, until now, no insert sequence including IS*Apl1* was found to involve in spread of *mcr-1* among all of the *mcr-1*-harbouring IncX4 plasmids. Thus, our aim is to explore the mechanism whereby the *mcr-1* gene is integrated into IncX4 plasmids.

In this work, we detect the presence of IncX4 plasmids among 2,470 Enterobacteriaceae isolates and concentrated on the *mcr-1*-positive IncX4 plasmids. Three representative IncX4 plasmids were subjected to high-throughput sequencing. We discussed the possible mode/pattern for the integration of *mcr-1* into IncX4 plasmids.

## Results

### Detection of IncX4 plasmids amongst Enterobacteriaceae

In total, forty-three of the 2,470 Enterobacteriaceae isolated from 2004 to 2013 were determined to possess IncX4 plasmids, including 23 from pigs, 12 from poultry, 3 from pets, and 3 from retail meat. The 43 IncX4-positive strains were collected from 10 different cities in Guangdong province, China. Following 16S-based identification of these IncX4-positive isolates, 41 species were assigned to *E. coli*, one isolate was classified into *K. pneumoniae*, and the remaining one was *Citrobacter freundii*.

### Molecular typing and antimicrobial susceptibility test of *mcr-1*-carrying strains

The PFGE-based genotyping showed that all the 41 IncX4-positive *E. coli* strains exhibited 31 different PFGE profiles, suggesting unexpected diversity amongst the *E. coli* host (Fig. S1). Among them, 13 were found to carry the *mcr-1* gene (Table 1). The 13 *E. coli* isolates are from pigs (n = 11) and pork (n = 2) between the years of 2007 and 2013. Of note, the two isolates (FEC46-4 and CEC49-3) possessed the identical PFGE profile and the other 11 isolates exhibited their own unique PFGE profiles, indicating that the 13 *mcr-1*-carrying *E. coli* isolates are epidemiologically unrelated (Fig. S1). Antimicrobial susceptibility tests revealed that all the *mcr-1*-positive *E. coli* isolates have higher MICs (4–8 μg/mL) in relative to *E. coli* 25922 (0.25 μg/mL). Furthermore, all of them were multidrug-resistant to ampicillin, nalidixic acid, olaquindox, tetracycline, florfenicol and sulfamethoxazole/trimethoprim. In addition, five of them were also resistant to extended-spectrum cephalosporins (ceftiofur and cefotaxime) (Table 1).

**Table 1:** Characteristics of the 13 *mcr*-positive *E. coli i*solates harbouring IncX4 plasmids.

Strain	PFGE	MIC (ug/ml)	Other resistance profile^a^	Resistance genes	RFLP pattern of variable region			Size of *mcr-1*-positive plasmid (kb)	*mcr-*positive plasmid transfer	PBRT		Genetic contextType^c^
		colistin			I	II	III			Wild strains^b^	conjugants	
FS1Z2S	X	8	AMP, CTX, CIF, CAZ, FOX, STR, GEN, KAN, NAL, CIP, OLA, FFL, TET, DOX, S*T	*mcr-1*, *oqxAB*	A	A	A	~33/90/320	+	X4, FII	X4	I
FS4Z2G	VIII	8	AMP, STR, GEN, KAN, AMK, NAL, CIP, OLA, FFL, TET, DOX, S*T	*mcr-1*, *oqxAB*, *rmtB*, *qepA*	A	A	A	~33/80	+	X4, FIB, FII	X4	I
FS2Z5C	XVII	8	AMP, KAN, NAL, CIP, OLA, FFL, TET, DOX, S*T	*mcr-1*, *oqxAB*	A	A	A	~33	+	X4	X4	I
FS11Z5C	VII	8	AMP, STR, GEN, KAN, NAL, CIP, OLA, FFL, TET, DOX, S*T	*mcr-1*, *oqxAB*	A	A	A	~33	+	X4, FIB, FII	X4	I
FZQ15-4-1	XXIV	8	AMP, FOX, GEN, KAN, NAL, CIP, OLA, FFL, TET, S*T	*mcr-1*	A	A	A	~33	+	X4	X4	I
CEC49-3	XX	4	AMP, CTX, CIF, STR, NAL, OLA, FFL, TET, DOX, S*T	*mcr-1*, *oqxAB*, *qnrS1*, *bla*_CTX-M-55,_	A	A	A	~33	−	X4, FIB, FII	−	I
FEC46-4	XX	4	AMP, CTX, CIF, STR, NAL, OLA, FFL, TET, DOX, S*T	*mcr-1*, *oqxAB*, *qnrS1*, *bla*_CTX-M-55_	A	A	A	~33	−	X4, FIB, FII	−	I
CSZ4	XXVIII	8	KAN, NAL, OLA, FFL, TET, DOX, S*T, CS	*mcr-1*, *oqxAB*, *qnrS1*	A	A	A	~33	+	X4	X4	I
QOC7-1	XI	8	AMP, STR, GEN, KAN, NAL, OLA, FFL, TET, DOX, S*T	*mcr-1*, *oqxAB*, *bla*_CTX-M-130,_	A	A	A	~33	+	X4, I2, FII	X4	I
PY1	XIV	8	AMP, NAL, FFL, TET, DOX, S*T	*mcr-1*	B	B	A	~33	+	X4	X4	II
FS170G	XXV	8	AMP, CTX, CIF, CAZ, FOX, STR, GEN, KAN, AMK, NAL, CIP, OLA, FFL, TET, DOX, S*T	*mcr-1*, *oqxAB*, *aac*(*6*′)*-Ib-cr*, *rmtB*	C	A	A	~33	+	X4, HI2, FIB	X4	III
S135	XVI	8	AMP, GEN, KAN, AMK, NAL, CIP, OLA, FFL, TET, DOX, S*T	*mcr-1*, *oqxAB*, *rmtB*	D	B	B	~60	+	X4, I2	I2	ND
FS13Z2S	XXII	8	AMP, CTX, CIF, STR, KAN, NAL, CIP, OLA, FFL, TET, DOX, S*T	*mcr-1*, *oqxAB*, *qnrS1*, bla_CTX-M−55_	E	C	C	~240/chromosome	+	X4, HI2	HI2	ND

^a^AMP, Ampicillin; CTX, cefotaxime; CIF, ceftiofur; FOX, cefoxitin; STR, streptomycin AMK, amikacin; GEN, gentamicin; KAN, kanamycin; NAL, nalidixic acid; CIP, ciprofloxacin; OLA, olaquindox; FLF, florfenicol; TET, tetracycline; DOX, doxycycline; S*T, sulfamethoxazole/trimethoprim; CS, Colistin; ^b^“−”, transfer of *mcr-1* was failure in strains CEC49-3 and CEC49-3; ^c^“ND”, not detected.

### Location and transferability of *mcr-1*

Thirteen *E. coli* isolates that both carried *mcr-1* and IncX4 plasmid were analyzed by S1-PFGE. The results showed that multiple plasmids, besides IncX4, were present in all of the 13 isolates (Fig. S2a). Southern blotting revealed that all the *mcr-1* harbouring isolates carried IncX4 plasmids of ~33 kb (Fig. S2b). The *mcr-1* gene was located on IncX4 plasmids in 11 isolates, or on a ~60 kb IncI2 plasmid and a ~240 kb IncHI2 plasmid in strain S135 and FS13Z2S, respectively (Table 1 and Fig. S2c). Of note, in strain FS1Z2S and FS4Z2G, besides IncX4 plasmid, the *mcr-1* gene was also observed on the other plasmid with ~90 kb and ~320 kb, as well as ~80 kb, respectively. In strain FS13Z2S, besides the copy on the non-IncX4 plasmid, a second copy of *mcr-1* was also found in the chromosome. The results indicate that multiple copies of *mcr-1* could exist in one isolate (Table 1 and Fig. S2c). Conjugation assay showed that *mcr-1* was successfully transferred in 11 out of the 13 isolates except CEC49-3 and FEC46-4. All the transconjugants showed 32- or 64-fold increases in the MICs of colistin, when compared with the recipient *E. coli* C600 (0.125 mg/L). However, the other antibiotic-resistant phenotypes did not co-transfer with colistin except for strain FS13Z2ST that was not only resistant to colistin but also to multi-drugs including ampicillin, nalidixic acids, tetracycline, florfenicol, cefotaxime, ceftiofur (Table 1).

### Aanalysis of *mcr-1*-harbouring IncX4 plasmids and Sequencing

**Figure 1:**
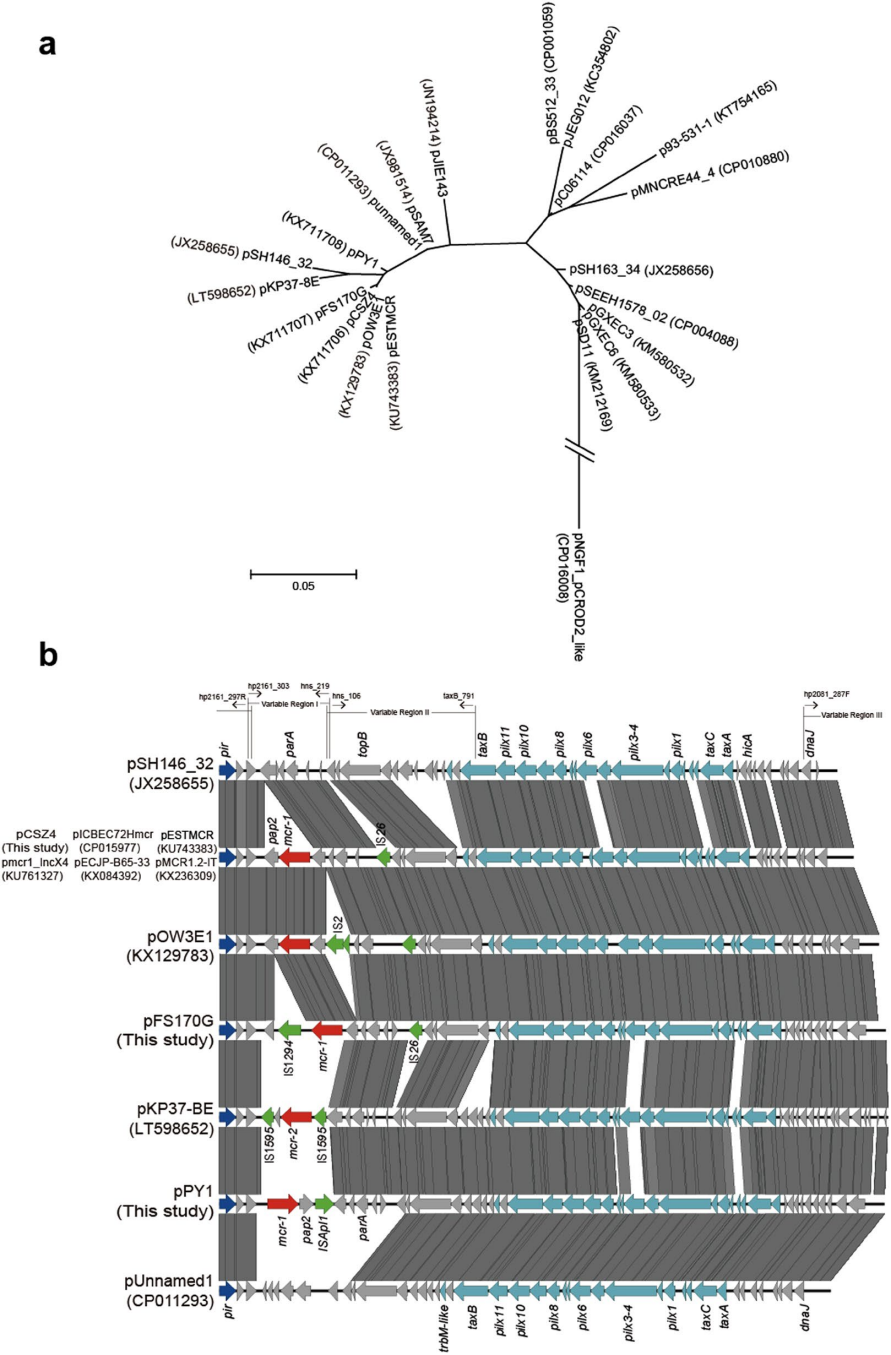
Phylogenetic tree and linear comparison of the 21 IncX4 plasmids. A total of 29 plasmids were collected by July 18, 2016, but only one was chosed for analysis in the highly similar plasmids. These plasmids included 3 found in this study and 18 downloaded from the GenBank (Table S1). (**a**) The trees are generated using MEGA (version 6) based on the complete sequence of IncX4 plasmids. (**b**) Boxed arrows represent the position and transcriptional direction of ORFs. Regions of >99% identity are marked by grey shading. Genes associated with the *tra* and *pil* loci are colored light blue, replication associated genes are colored dark blue, antibiotic resistance genes are colored red, insertion sequences are colored green, and other genes are colored gray. The Variable Region I, II, and III, as well as the primers used were marked.

We compared the regions surrounding *mcr-1* occurred between the *pir* and *hns* genes in all of the 11 isolates harbouring *mcr-1*-IncX4 plasmids, three different genetic contexts were found (Fig. S3). Three representative *mcr-1-*harbouring IncX4 plasmids pCSZ4, pFS170G and pPY1 were obtained and submitted to be sequenced. They were 33.309 kb, 34.924 kb, and 34.99 kb in length with GC content of 41.85%, 41.56%, and 42.48%, respectively. Three plasmids are nearly identical, only having subtle differences within the resistance region. All of them belong to IncX4 type plasmids and have typical plasmid backbones set that are responsible for plasmid replication, maintenance, and transfer. The phylogenetic tree revealed two distinct clusters: I and II (Fig. 1a). All the *mcr-1*-harbouring IncX4 plasmids belonged to the cluster I, which had a *pir*-type replicon. In the previous studies, the *pir*-type plasmids carrying *bla*_CTX-M_ were identified in *E. coli* from the United Kingdom^8^ and Australia^32^ (Fig. 1a). Further comparative analysis indicated that plasmid pCSZ4 was nearly identical to IncX4 *mcr-1*-harbouring *E. coli* plasmids pECJP-B65-33 (Accession no.: KX084392) isolated from China, pICBEC72Hmcr (Accession no.: CP015977) from Brazil, pESTMCR (Accession no.: KU743383) from Estonia, and *K. pneumoniae* plasmids pMCR1_Incx4 (Accession no.: KU761327) from China and pMCR1.2-IT (Accession no.: KX236309) identified in Italy (Fig. 1b and Table S1).

Based on the sequence comparison of the 29 IncX4 plasmids deposited in Genbank database, three major variable regions of these IncX4 plasmids were identified (Fig. 1b). Three pairs of specific primers were designed for the variable regions and used to type the regions in the plasmid IncX4 and *mcr-1* positive *E. coli* isolates by PCR-RFLP (Fig. S4). Five (designated A–E), three (designated A–C), and three (designated A–C) patterns (each with 100% similarity) were identified in the Variable Region I, II and III, respectively. Five different profiles for IncX4 plasmids were found by combination of the PCR-RFLP band patterns of the three major variable regions in the 13 isolates, and one profile (designated A-A-A) was predominant (9 out of 13) (Table 1). The PCR products of each variable region were randomly selected for sequence determination to represent all the distinct patterns in every region. Interestingly, the analyses of these sequences showed that the *mcr-1* gene located on the Variable Region I (represented by patterns A, B and C) of 11 isolates (Table 1), and no other resistance genes were found in the three variable regions.

### Comparison of the region containing *mcr-1* on IncX4 plasmids

**Figure 2:**
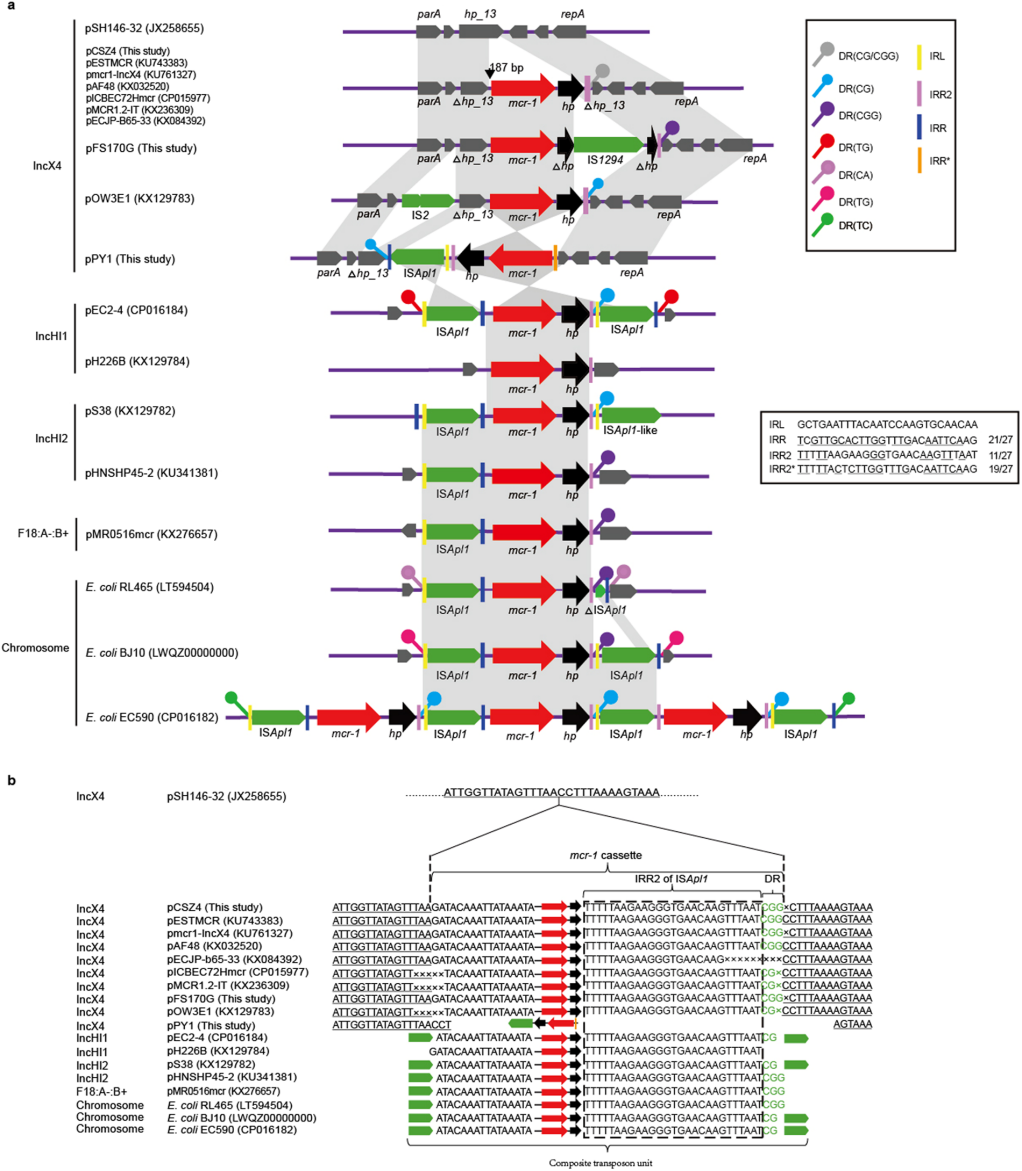
Contexts of *mcr-1* on IncX4 plasmids and relevant the other plasmids and *E. coli* chromosomes. Resistance genes are indicated by red arrows, while accessory genes are indicated by gray arrows. Insertion sequences are highlighted in green arrows labelled with their name or number. DRs are represented by the filled circles. The short black arrow show the 187 bp spacer between IS*Apl1* and *mcr-1*. Vertical black bars represent the transposon IR of IS*Apl1*, while the dotted lines indicate possible deletion and insertion events. DR, direct repeats. IRL, terminal inverted repeats of left. IRR, terminal inverted repeats of right. Underlined nucleotides in the alternate IRR elements are identical to those of the perfect IRR. The underlined bases is the backbone of pSH146-32. The big brace is the *mcr-1* cassette. The bases in the box are IRRs, while the green bases are DRs. (**a**) Genetic environment of *mcr-1* on IncX4 plasmids and relevant the other plasmids and *E. coli* chromosomes. (**b**) Alignment of the *mcr-1* gene and its adjacent squence.

In comparison with pCSZ4, another two *mcr-1-*harbouring IncX4 plasmids pFS170G and pPY1 are different in the *mcr-1*-harbouring variable region. Like other *mcr-1*-harbouring IncX4 plasmids, in pCSZ4, only a typical *mcr-1* cassette encompassing the *mcr-1* gene and a hypothetical protein (*hp1*) was identified. In pFS170G, the *mcr-1* genetic contexts were identical to that in pCSZ4 except the flanked *hp* was truncated by IS*1294*. As for pPY1, the whole *mcr-1* cassette was inverted and IS*Apl1* was inserted directly downstream of the *hp*. Of note, in all the IncX4 plasmids, the insertion sequence IS*Apl1* was absent in front of the *mcr-1*. However, a proposed IRR (IRR2, TTTTTAAGAAGGGTGAACAAGTTTAAT) was consistently present on the 3′-region of *hp* (Fig. 2b). Moreover, 2 or 3 bases (CG or CGG) adjacent to the IRR2 were recognized as DRs which were characterized as the classic direct repeat sequence at the target insertion site along with the transposition of the IS*Apl1*. Intriguingly, compared to pSH146_32 without carrying *mcr-1*, nearly identical inserted site of the *mcr-hp* transposition unit was found in all *mcr-1*-located IncX4 plasmids except for pPY1, in which the inserted location appeared on 3 bases downstream of the stationary inserted site, as well as the transposition unit was completed inverted. Through further comparative analysis of the genetic environments of *mcr-1*, an identical IRR2 and DR were also found within IncHI1 plasmid (pEC2-4 and pH226B), IncHI2 plasmid (pS38 and pHNSHP45-2), F18:A−:B+ plasmid (pMR0516mcr) or *E. coli* chromosomes (*E. coli* RL465, *E. coli* BJ10 and *E. coli* EC590) (Fig. 2b).

## Discussion

Previous evidence revealed that IncX4 plasmids played a significant role in the spread of the *mcr-1* gene among Enterobacteriaceae^15, 26, 27^. In this study, *mcr-1* located on IncX4 plasmids in 11 of the 13 *mcr-1*-positive isolates. Moreover, nine among them belong to an epidemic pCSZ4-like IncX4 plasmid. Interestingly, the predominant *mcr-1*-carrying IncX4 plasmids were detected in the *E. coli* isolates from five different cities in South China between 2012 and 2013, but the isolates were epidemiologically unrelated (Fig. S1). Conjugation assay showed that most of IncX4 plasmids carrying *mcr-1* (9/11) were transferable (Table 1). The genetic analysis revealed that the Variable Region I in the pCSZ4-like plasmids was almost identical to that in the IncX4 plasmids of Enterobacteriaceae isolates (*E. coli* and *K. pneumoniae*) from countries of several continents (Fig. 1), suggesting that the predominant *mcr-1*-harbouring IncX4 plasmids have circulated in Enterobacteriaceae worldwide.

Multiple replicons such as IncHI2 and IncI2 were observed to co-exist with IncX4 in the *mcr-1-*carrying isolates. In FS1Z2S and FS4Z2G, *mcr-1* has two copies and separately located on two types of plasmids (Table 1). In addition, the *mcr-1* is also detected on an IncX4 plasmid and its chromosome in strain FS13Z2S. It is interesting that multiple copies of *mcr-1* are carried by co-resident plasmids or chromosome in one strain (Fig. S2). However, whether the emergence of two copies of *mcr-1* in a single strain is due to transposition of *mcr-1* cassette between plasmids and/or chromosome is still unknown.

Generally, the IS*Apl1* insertion sequence is detected upstream of *mcr-1* and it is proposed to involve in the mobilization of the *mcr-1* cassette^30^. Recent reports provided evidence that *mcr-1* was mobilized primarily as a composite transposon composed of copies of IS*Apl1* or through formation of a circular intermediate^31, 33^. But we noticed that many sequences franked with only one or no IS*Apl1*, and the 3′ end of *mcr-1*-*hp1* unit was flexible. Snesrud *et al.* explained that it lost one or both copies of IS*Apl1* after composite transposon^31^. They attributed the differences at the 3′ end of *mcr-1*-*hp1* unit to IS*Apl1* partially or completely removed by a process that generated mismatches and deletion^31^. We thought the above model for transposition of the *mcr-1* gene could not fully explain the phenomenon. IS*Apl1* is a member of the IS*30* family, which may format circular intermediates during transposition by recognizing its own IRL and the closest downstream sequence that resembles its IRR^34^. Further analyses of the sequences carrying *mcr-1*, we found that the 3′ end of *mcr-1*-*hp1* could match with the perfect IRR of IS*Apl1* “coincidently”, which was named as IRR2 in this study (Fig. 2). Here, a new potential linked transposition model was proposed in *mcr-1*-harbouring sequences that initiated at the 27 bp IRL sequence of IS*Apl1*, but ended at a fixed position downstream of the *mcr-1* by recognizing the related IRR. Insertion of an IS*Apl1* results in the duplication at the target insertion site of two or three base pairs^35^, which could be recognized as a “relic” to track an insertion event.

Although the IS*Apl1* is lost in front of *mcr-1* on all the *mcr-1*-harbouring IncX4 plasmids, several evidences supported the new mode we proposed above: (1) a 187 bp inter-genic region between IRR of IS*Apl1* and replication initiator of *mcr-1* is consistently remained except that 6 bp is deleted in three plasmids including the *mcr-*1.2-harbouring plasmid, pMCR1.2-IT. (2) a flexible IRR-like sequence (IRR2, TTTTTAAGAAGGGTGAACAAGTTTAAT), sharing 11/27 bp similarity with the perfect IRR, is steadfastly identified on the 3′region of hp. (3) a suspected DR exactly emerges neighboring the IRR2 (Fig. 2). These evidences show that in IncX4 plasmid the IS*Apl1* is probably linked with the transposition of the *mcr-1* cassette like which in the paradigm *mcr-1*-containing plasmid pHNSHP45, and it is subsequently lost due to some unknown event. Loss of IS*Apl1* seems to be conducive to maintaining of *mcr-1* on IncX4 plasmids. Whether it is more beneficial to the host bacteria to adapt to changed environments, especially a conversion from the pressure of antimicrobial agents to a pressure-free environment, thereby avoiding the resistance region lost need to be evaluated in future studies^36^. Of note, the other plasmids such as IncHI1, IncHI2, IncF18:A−:B+, as well as *E. coli* chromosomes share the same IRR2 and DR with that in IncX4 plasmids (Fig. 2b), implying they may have a common ancestor.

In conclusion, genetic analyses revealed that an epidemic *mcr-1*-harbouring IncX4 plasmid might circulate in Enterobacteriaceae of diverse origins worldwide. IS*Apl1*-mediated transposition by recognizing different related IRRs probably involved in mobilization of the *mcr-1*-*hp1* unit into the IncX4 plasmids. Our findings also demonstrate that most of the IncX4 plasmids along with the other IncHI1, IncHI2, IncF18:A−:B+ plasmids and even *E. coli* chromosomes may have acquired *mcr-1* genes by a common mechanism.

## Materials and Methods

### Bacterial isolates and detection of IncX4 plasmids

Totally, 2,470 Enterobacteriaceae isolates were screened for the presence of IncX4 plasmids by PCR with the specific primers earlier reported^3^. The isolates were obtained from food animals (n = 1,044 from pigs and n = 722 from birds), companion animals (n = 381), and retail meat (n = 323) during 2002–2013 in Guangdong province in South China. Among them, 1,766 food animal isolates were sampled from viscera or fecal samples of diseased or healthy animals from farms during 2002–2013; and 381 pet strains were randomly collected from feces, urine, pus or sneeze samples from pet hospitals during 2008–2012. Strains of animal product origins were randomly collected from fresh pork (n = 233), chicken (n = 75), beef (n = 10) and duck (n = 5) from commodities markets and supermarkets in 2012. The identities of the IncX4-positive isolates were confirmed by 16S rDNA sequencing and MALDI-TOF MS (Biomerieux, France). IncX4-positive isolates were further analyzed by PFGE using enzyme *Xba* *I* ^37^. Comparison of PFGE patterns was performed by using BioNumerics software version 2.5 (Applied Maths), and clusters were defined by cutoff of 85% similarity between DNA patterns.

### PCR screen for the *mcr-1* gene and antimicrobial susceptibility tests

All of the IncX4-positive isolates were subjected to PCR-screen for the *mcr-1* gene with the primers described previously^9^. In total, 19 antibiotics were tested here (ampicillin, cefoxitin, ceftiofur, cefotaxime, amikacin, gentamicin, kanamycin, streptomycin, florfenicol, doxycycline, tetracycline, nalidixic acid, ciprofloxacin, olaquindox, sulfamethoxazole/trimethoprim, meropenem, colisin, fosfomycin and tigecycline). The minimum inhibitory concentration (MIC) of various antibiotics was determined by the agar dilution method following the guidelines of Clinical and Laboratory Standards institute (CLSI). The EUCAST breakpoints for *E. coli* were applied for colistin and tigecycline^38^. The breakpoints for other antimicrobial were used as recommended by the CLSI (M100-S25) or CLSI (Vet01-A4/Vet01-S2)^39, 40^. *E. coli* ATCC 25922 was used as a control.

### Location of the *mcr-1* gene and conjugation assay

To determine the association of the IncX4 plasmid and the *mcr-1* gene, all IncX4 plasmids harbouring *mcr-1* were analyzed by S1-PFGE and Southern blotting with the digoxigenin-labeled probes (Roche Diagnostics GmbH, Germany) specific for the *taxC* and *mcr-1* genes^30, 41^. Furthermore, the transferability of the *mcr-1* gene was assessed in all the *mcr-1*-carrying isolates by filter mating using streptomycin-resistant *E. coli* C600 as a recipient. Briefly, donor bacterium and recipient was grown in Luria Bertani Broth (LB) to logarithmic phase, mixed at a 1:4 ratio (vol/vol), collected in a filter, and incubated at 37 °C for 20 h. Transconjugants were selected on Eosin-methylene blue agar plates supplemented with streptomycin (2000 µg/mL) and colistin (2 µg/mL). The transconjugants acquiring the *mcr-1* gene were confirmed by both PCR and antimicrobial susceptibility test. Incompatibility (Inc) groups were assigned by PBRT and the revised IncX typing procedure for the wild isolates and their transconjugants^3, 5, 42^.

### Aanalysis of *mcr-1*-harbouring IncX4 plasmids and Sequencing

One pair of specific primers for amplification of the region between *pir* (replicon protein) and *hns* (DNA-binding protein) was designed to explore the genetic contexts of *mcr-1* on IncX4 plasmids. *Mcr-1*-harbouring IncX4 plasmids with different genetic contexts were selected and then prepared from the transconjugants using the QIAGEN Plasmid Midi kit (QIAGEN) and were sequenced by Illumina MiSeq technique (Illumina, San Diego, USA). Illumina sequences were *de novo* assembled using SOAP *de novo* ^43^. The gaps between the contigs were closed by PCR and respective amplicons were sequenced. Gene prediction and annotation were performed using the RAST tools^44^. To gain insights into the variations of IncX4 plasmids, sequence comparisons of the 30 completely-sequenced IncX4 plasmids (collected until July 18, 2016) were applied BLAST and Easyfig^45^. The variable regions of the other IncX4 plasmids without sequencing in this study were further analyzed by PCR-RFLP. The PCR products of the variable regions were purified and then digested with the following restriction enzymes (TaKaRa, Dalian, China): *ClaI* for Variable Region I, *EcoRV* for variable Region II, and *HincII* for Variable Region III. Comparison of PCR-RFLP patterns were performed with BioNumerics software version 2.5 (Applied Maths), and clusters were defined by cutoff of 100% similarity between DNA band patterns.

### Nucleotide sequence accession numbers

The complete nucleotide sequences of pCSZ4, pFS170G and pPY1 have been deposited into GenBank database under the accession numbers KX711706, KX711707, and KX711708, respectively.

### Ethics Statement

This study protocol was approved by the South China Agriculture University Animal ethics committee and carried out in accordance with relevant guidelines. The owners of the farm animals and companion animals from which faecal swabs were taken gave permission for their animals to be used in this study.

## Electronic supplementary material

Supplementary Materials
