# Supplementary material for: Genetic Analysis of the IncX4 Plasmids: Implications for a Unique Pattern in the mcr-1 Acquisition
Source: Sci Rep. 2017 Mar 24;7:424. doi: 10.1038/s41598-017-00095-x (PMC5428312; doi:10.1038/s41598-017-00095-x)
Supplement: Supplementary file 1 — Supplementary Materials [file 41598_2017_95_MOESM1_ESM.doc]

**Supplementary Information for**

**Title:** Genetic Analysis of the IncX4 Plasmids: Implications for a Unique Pattern in the *mcr-1* Acquisition

**Authors:** Jian Sun^a,b#^, Liang-Xing Fang^a,b#^, Zuowei Wu^c^, Hui Deng ^a,b^, Run-Shi Yang^a,b^,, Xing-Ping Li^a,b^, Shu-Min Li^a,b^, Xiao-Ping Liao^a,b^, Youjun Feng^d^, and Ya-Hong Liu^a,b*^

**Affiliations:**

^a^ National Risk Assessment Laboratory for Antimicrobial Resistance of Animal Original Bacteria, South China Agricultural University, Guangzhou, P. R. China.

^b^ Guangdong Provincial Key Laboratory of Veterinary Pharmaceutics Development and Safety Evaluation, South China Agricultural University, Guangzhou, P. R. China.

^c^ Department of Veterinary Microbiology and Preventive Medicine, College of Veterinary Medicine, Iowa State University, USA.

^d^ Department of Medical Microbiology and Parasitology, Zhejiang University School of Medicine, Zhejiang 310058, P. R. China

^*^To whom correspondence should be addressed: [lyh@scau.edu.cn](mailto:lyh@scau.edu.cn) or fengyj@zju.edu.cn

**Supplementary Tables**

**Table S1:** Characteristics of the 29 IncX4 plasmids of complete nucleotide sequence in GenBank (collected by July 18, 2016).

**Table S2:** Oligonucleotide primers and restriction endonucleases used for PCR-RFLP analysis of variable regions of IncX4.

Table S1 Characteristics of the 29 IncX4 plasmids of complete nucleotide sequence in GenBank (collected by July 18, 2016).

| plasmids | Size (bp) | Species ^a^ | Source ^b^ | Country | Resistance genes | Variable Region^c^ | Accession No. |
| --- | --- | --- | --- | --- | --- | --- | --- |
| pSEEH1578_02 | 35297 | *S. enterica* | Human | USA | No | -^d^ | CP004088 |
| pCROD2 | 39265 | *C. rodentium* | Human | USA | No | - | FN543504 |
| pJIE143 | 34345 | *E．coli* | Human | Australia | *bla*_CTX-M-15_ | III | JN194214 |
| pSAM7 | 35341 | *E．coli* | Cattle | UK | *bla*_CTX-M-14b_ | NT^e^ | JX981514 |
| pBS512_33 | 33103 | *S. boydii* | ND | USA | No | - | CP001059 |
| pSH696_34 | 33765 | *S. enterica* | Turkey | USA | No | - | JX258654 |
| pSH146_32 | 32447 | *S. enterica* | Porcine | USA | No | - | JX258655 |
| pSH163_34 | 33763 | *S. enterica* | Turkey | USA | No | - | JX258656 |
| pJEG012 | 41919 | *K. pneumoniae* | Human | Australia | *aacA4*, *bla*_OXA-9_, *bla*_TEM-1_ *bla*_CTX-M-15_ | I | KC354802 |
| pSD11 | 37672 | *E．coli* | Swine | China | *cfr* | I | KM212169 |
| pGXEC6 | 38405 | *E．coli* | Swine | China | *cfr* | I | KM580533 |
| pGXEC3 | 41646 | *E．coli* | Swine | China | *cfr* | I | KM580532 |
| pMNCRE44_4 | 30855 | *E．coli* | Human | USA | No | - | CP010880 |
| pESTMCR | 33311 | *E．coli* | Pig | Estonia | *mcr-1* | I | KU743383 |
| p93-531-1 | 34449 | *S. dysenteriae* | ND | France | *dfrA1* | II | KT754165 |
| pmcr1_IncX4 | 33287 | *K. pneumoniae* | Human | China | *mcr-1* | I | KU761327 |
| pAF48 | 31808 | *E．coli* | Human | South Africa | *mcr-1* | I | KX032520 |
| pMCR1.2-IT | 33303 | *K. pneumoniae* | Human | Italy | *mcr-1.2* | I | KX236309 |
| pICBEC72Hmcr | 33304 | *E．coli* | Human | Brazil | *mcr-1* | I | CP015977 |
| pOW3E1 | 34640 | *E．coli* | River water | Switzerland | *mcr-1* | I | KX129783 |
| pECJP-B65-33 | 33298 | *E．coli* | Pig | China | *mcr-1* | I | KX084392 |
| pUnnamed1 | 32098 | *S. enterica* | Human | Germany | No | - | CP011293 |
| pUnnamed1 | 32098 | *S. enterica* | Human | Germany | No | - | CP011290 |
| pNGF1_pCROD2_like | 40158 | *E．coli* | Mice | USA | No | - | CP016008 |
| pKP37-BE | 35104 | *E．coli* | porcine | Belgium | *mcr-2* | I | LT598652 |
| pC06114_3 | 33059 | *E．coli* | ND | Germany | No | - | CP016037 |
| pCSZ4 | 31229 | *E．coli* | Pork | China | *mcr-1* | I | KX711706 |
| pFS170G | 32917 | *E．coli* | Swine | China | *mcr-1* | I | KX711707 |
| pPY1 | 32864 | *E．coli* | Pork | China | *mcr-1* | I | KX711708 |

^a^*E．coli*, *Escherichia coli*; *S. enterica*, *Salmonella enterica*; *K. pneumonia,* *Klebsiella pneumonia*; *S. boydii*, *Shigella boydii*; *S. dysenteriae*, *Shigella dysenteriae*; *C. rodentium*; *Citrobacter rodentium*.^b^“ND”, not determined; ^c^ to determine the three variable regions of IncX4 plasmids where the resistance genes was located according to this study. ^d^“-”, not detected. ^e^“NT”, *bla*_CTX-M-14b_ was not located in the three major variable regions.

Table S2 Oligonucleotide primers and restriction endonucleases used for PCR-RFLP analysis of variable regions of IncX4 plasmids in this study

| Target | Primer name | Nucleotide sequence (5’ to 3’) | Amplicon | |
| --- | --- | --- | --- | --- |
|  |  |  | Position^a^ | Endonuclease |
| Variable Region I | hp2161_303 | TGAAGTGCAGAATATAACCTGT | 8586..15083 | *ClaI* |
|  | hns_219 | TTTAGGTCTTGATCCCGAAT |  |  |
| Variable Region II | hns_106 | CCATTCTCTGATCTCGCCAT | 14951..24316 | *EcoRV* |
|  | taxB_791 | CACCGTTTGCTAACCCCAA |  |  |
| Variable Region III | hp2081_287F | TCACCGTAATTATATCGTGCAT | 3541..8597 | *HincII* |
|  | hp2161_297R | TTCTGCACTTCACGGATT |  |  |

^a^GenBank accession number FN543504

**Supplementary Figure**

**Fig.S1** Dendrogram to illustrate the genetic relatedness of the 41 IncX4-positive *E. coli* strains from diverse origins by pulsed-field gel electrophoresis (PFGE).

**Fig.S2** Location of the *mcr-1* gene in 13 *E. coli* strains harbouring *mcr-1*. (a) S1 nuclease-PFGE. (b) Southern blotting, hybridization with the *taxC* gene probe and (c) *mcr-1.* Lanes 1-13, FZQ15-2-1, CSZ4, FEC46-4, CEC49-3, QOC7-1, FS2Z5C, FS13Z2S, FS1Z2S, FS11Z5C, FS4Z2G, PY1, S135, FS170G.

**Fig S3** Characteristics of the genetics contexts of mcr-1 found in this study.

**Fig.S4** Dendrogram to illustrate the restriction fragment length polymorphism

(RFLP) of the three major variable regions of the IncX4 plasmids in 13 *E. coli* strains harboring *mcr-1* in this study.

**
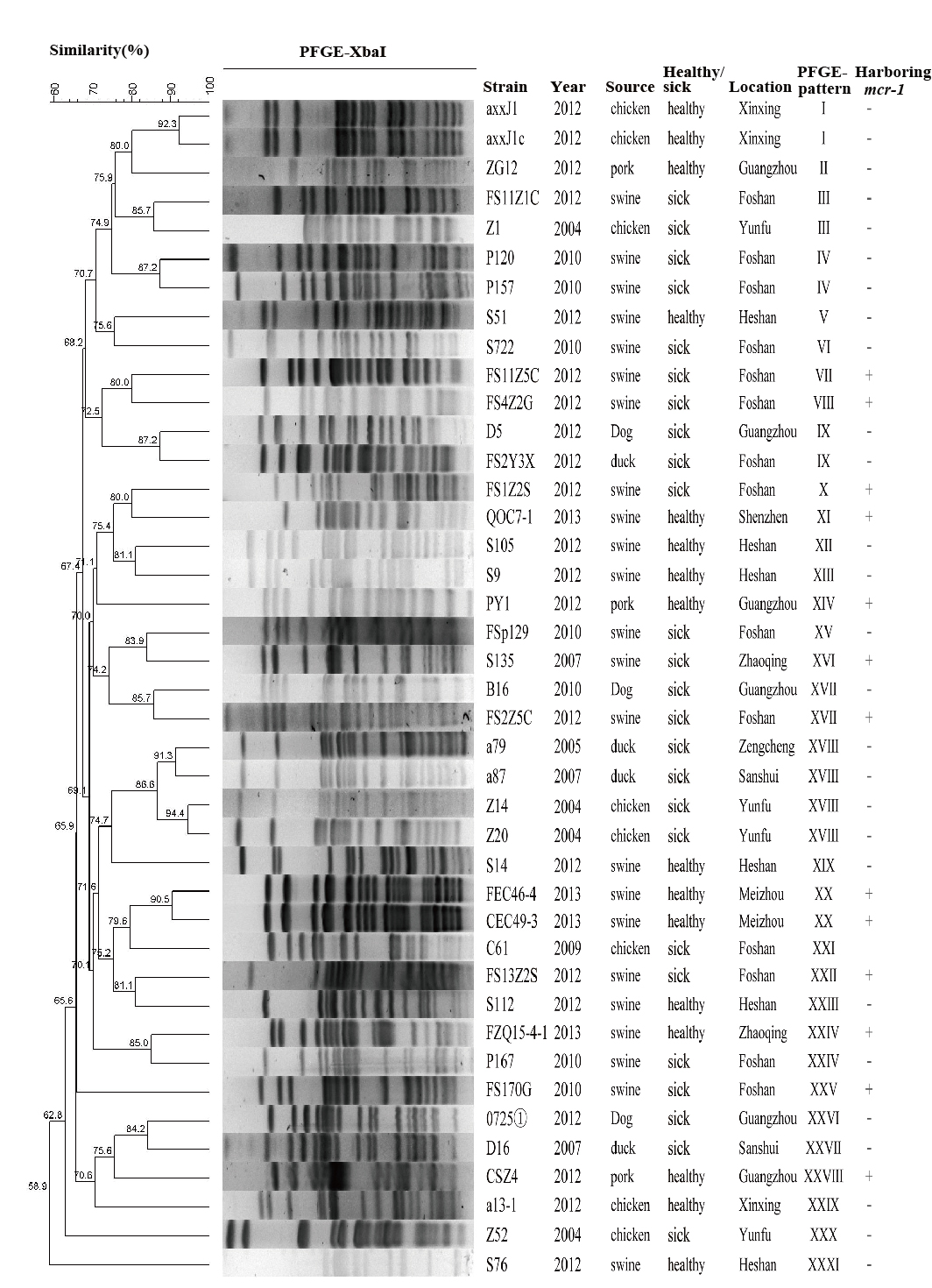
**

**
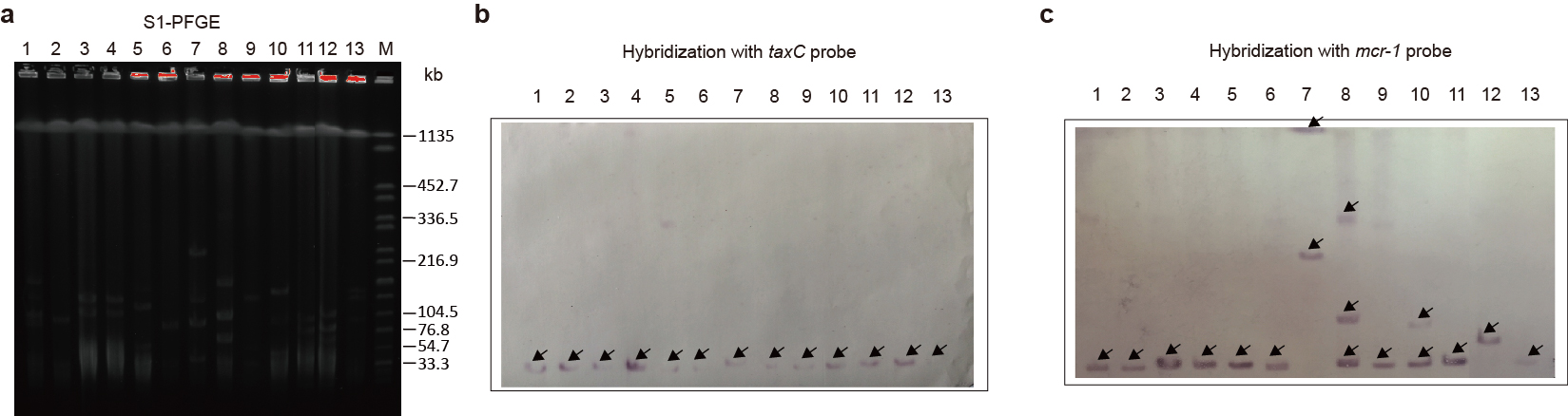
**

**
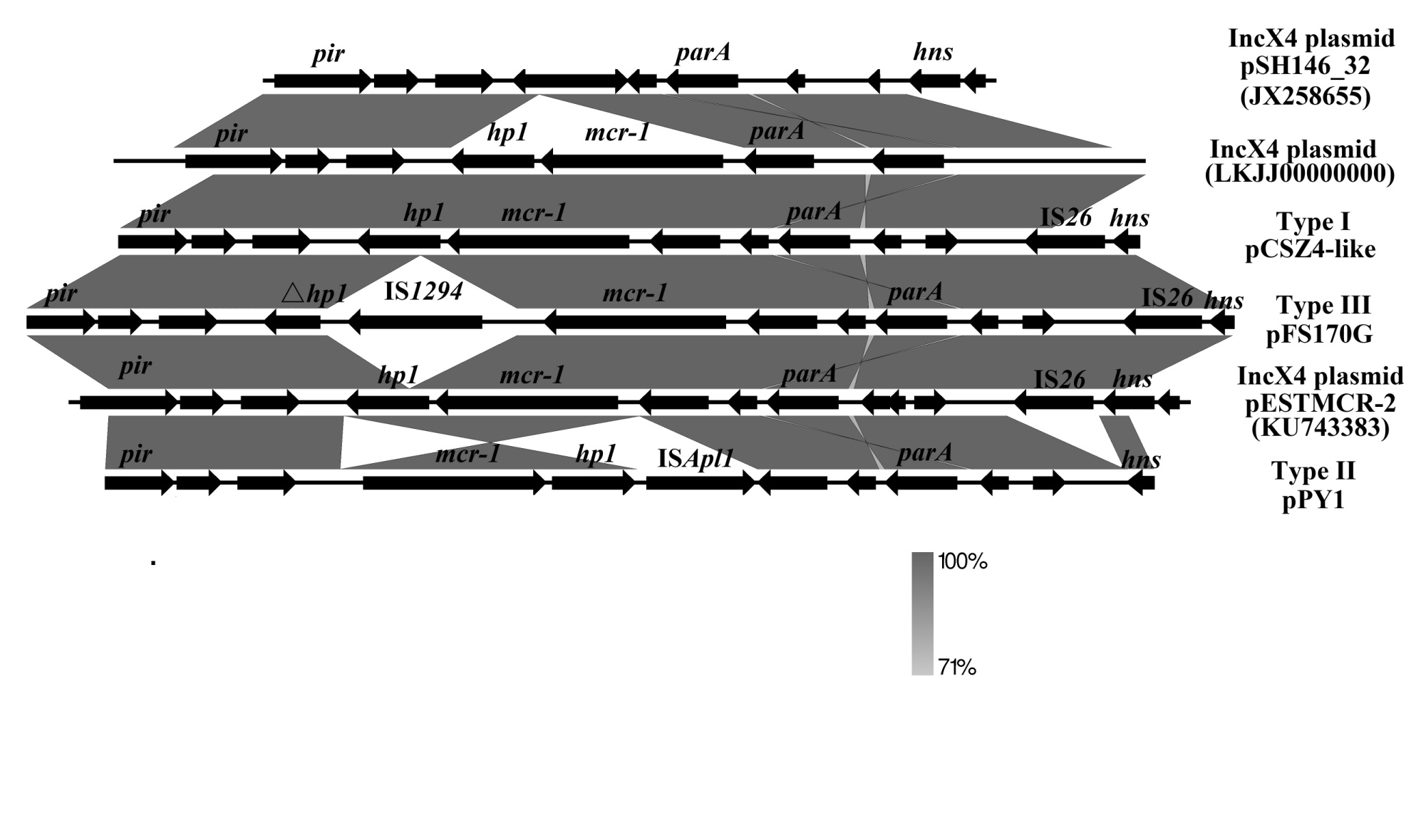
**

**
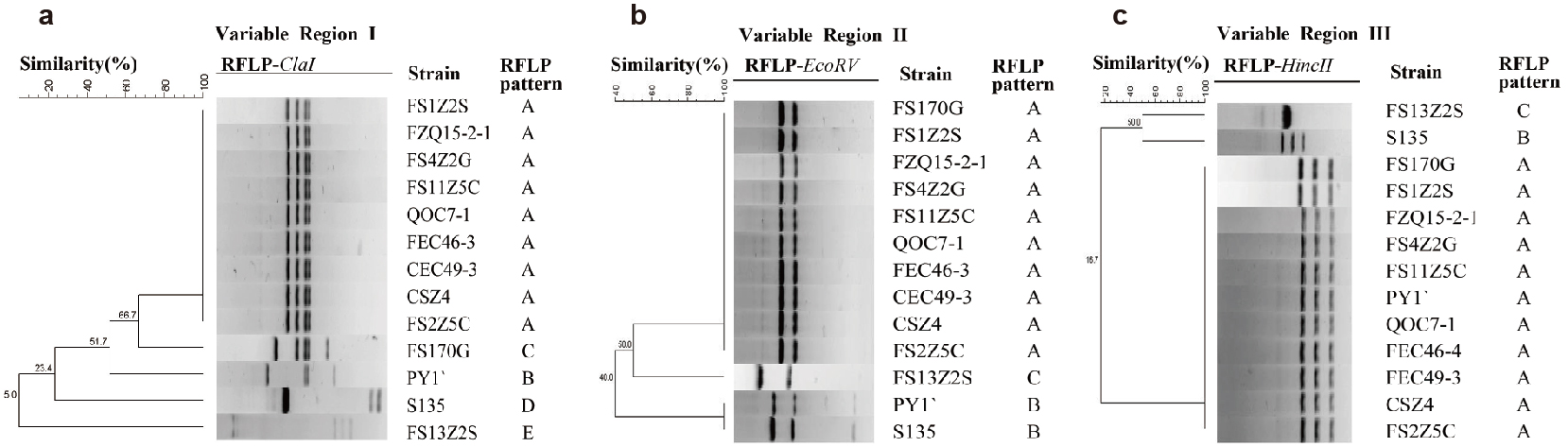
**
